# Promoting the translation of intentions into action by implementation intentions: behavioral effects and physiological correlates

**DOI:** 10.3389/fnhum.2015.00395

**Published:** 2015-07-14

**Authors:** Frank Wieber, J. Lukas Thürmer, Peter M. Gollwitzer

**Affiliations:** ^1^Social Psychology and Motivation Group, Department of Psychology, University of KonstanzKonstanz, Germany; ^2^Graduate School of Decision Sciences, University of KonstanzKonstanz, Germany; ^3^Motivation Lab, Department of Psychology, New York University, New YorkNY, USA

**Keywords:** implementation intentions, goals, action, self-regulation, EEG, fMRI

## Abstract

The present review addresses the physiological correlates of planning effects on behavior. Although intentions to act qualify as predictors of behavior, accumulated evidence indicates that there is a substantial gap between even strong intentions and subsequent action. One effective strategy to reduce this intention–behavior gap is the formation of implementation intentions that specify when, where, and how to act on a given goal in an if-then format (“If I encounter situation Y, then I will initiate action Z!”). It has been proposed that implementation intentions render the mental representation of the situation highly accessible and establish a strong associative link between the mental representations of the situation and the action. These process assumptions have been examined in behavioral research, and in physiological research, a field that has begun to investigate the temporal dynamics of and brain areas involved in implementation intention effects. In the present review, we first summarize studies on the cognitive processes that are central to the strategic automation of action control by implementation intentions. We then examine studies involving critical samples with impaired self-regulation. Lastly, we review studies that have applied physiological measures such as heart rate, cortisol level, and eye movement, as well as electroencephalography (EEG) and functional magnetic resonance imaging (fMRI) studies on the neural correlates of implementation intention effects. In support of the assumed processes, implementation intentions increased goal attainment in studies on cognitive processes and in critical samples, modulated brain waves related to perceptual and decision processes, and generated less activity in brain areas associated with effortful action control. In our discussion, we reflect on the status quo of physiological research on implementation intentions, methodological and conceptual issues, related research, and propose future directions.

## Introduction

“The road to hell is paved with good intentions" is a popular proverb that illustrates a potential obstacle during action control: people may form adequate intentions but fail to translate these intentions into action. Indeed, research has observed a pervasive gap between people’s intended and actual behavior (Ajzen, 1991, 2012; Armitage and Conner, 2001; Sheeran, 2002). Examples of such intention–behavior gaps can be found in various domains including intentions to exercise more (meta-analysis by Bélanger-Gravel et al., 2013) or to eat more healthily (meta-analysis by Adriaanse et al., 2011b), intentions to be more careful with the disclosure of personal information during interpersonal communication (Norberg et al., 2007), and intentions to make ethical purchases (Carrington et al., 2014).

### From Intention to Action

Humans regulate their behavior by setting goals and then acting toward them (overview by Fishbach and Ferguson, 2007). The field of social and personality psychology as well as the field of human cognitive neuroscience both define goals as mental representations of desired endstates (Braver et al., 2014). While the former domain emphasizes considerations of feasibility, desirability, commitment, and beliefs, the latter focuses on the active maintenance of these representations. Aside from these different accentuations, the two research fields thus share a similar conceptual starting point that allows the comparison and integration of behavioral and physiological findings on the intentional control of action.

To investigate the causal impact of intentions on action, Webb and Sheeran (2006) conducted a meta-analysis of behavioral experiments that manipulated the strength of the intention (i.e., induced either high or low goal commitment). Because participants were assigned randomly to one of these conditions, these research designs make third-variable explanations highly unlikely, allowing causal conclusions to be drawn. The analysis revealed that a medium-to-large (Cohen, 1992) change in commitment (*d* = 0.66) only led to a small-to-medium change in the respective behavior (*d* = 0.33), suggesting that forming strong intentions (i.e., forming high goal commitment) is not sufficient for goal attainment. A related meta-analysis showed that this gap is largely due to people who are strongly committed but fail to act on their intentions (Sheeran, 2002). Consequently, there is a gap between strong intentions and actual behavior such that even strong goal intentions do not ensure successful goal attainment.

### Planning with Implementation Intentions: Effects and Processes

An effective and easily applicable self-regulation strategy for supporting people in their efforts to translate goal intentions into action is planning when, where, and how they want to act toward a goal in an if-then format (e.g., “If I encounter situation Y, then I will initiate action Z!”). Forming such implementation intentions (Gollwitzer, 1993, 1999, 2014) supports individuals (Gollwitzer and Sheeran, 2006) as well as groups (Wieber et al., 2012, 2013; Thürmer et al., 2015a) in their efforts to reduce their intention-behavior gaps. For instance, the meta-analysis by Gollwitzer and Sheeran (2006) involving more than 8,000 participants in 94 independent studies observed that implementation intentions have a medium-to-large average effect on goal attainment (*d*= 0.65). This is especially remarkable as most of the included studies compared an implementation intention condition to a control condition that formed goal intentions, which already benefit goal attainment (Webb and Sheeran, 2006).

The effectiveness of implementation intentions is assumed to be based on two processes (Gollwitzer, 1999; overview by Gollwitzer and Oettingen, 2011). First, the mental representation of the situation specified in the if-part becomes highly accessible once the respective goal is activated. Second, a strong associative link between the mental representation of the situation and the mental representation of the action specified in the then-part is established. As a result, individuals should show heightened perceptual readiness regarding the critical situation and should therefore initiate the linked action efficiently, immediately, and without requiring further conscious intent. Metaphorically speaking, forming an if-then plan thus strategically automates goal striving by shifting from top–down to bottom–up information processing. People intentionally delegate control of goal-directed behavior to preselected situational cues, with the explicit purpose of attaining their goals.

## The Present Review

In the present review, we first report studies on the cognitive processes. As there is considerable overlap between the paradigms used in these behavioral studies on cognitive processes and those applied in physiological studies, these studies imply new physiological hypotheses and can inspire the design of new physiological studies. Subsequently, we review behavioral studies that have examined critical samples with self-regulation impairments, as these studies can also inform research on the cognitive and physiological processes underlying implementation intention effects. We then present studies using physiological measures that exemplify how physiological measures including cortisol level, heart rate, and eye movement recordings as well as electrocortical and neuroimaging measures can contribute to our understanding of implementation intention effects.

Although we aim to provide a comprehensive review, a complete review of the large number of implementation intention studies is beyond the scope of this review. We have selected studies that we believe are central to the implementation intention literature and methodologically as well as conceptually most informative for physiological research (see **Table 1**). In order to include as many studies as possible in a limited amount of space, we only describe exemplary studies in detail. In the first two sections on cognitive processes and critical samples, we briefly summarize this research and refer to the relevant literature. As physiological processes represent the focus of the present review and as the number of related implementation intention studies to date is small, in the third section we comprehensively describe all of the implementation intention studies using physiological measures that we are aware of.

**Table 1:** Overview of the studies featured in the present review.

Study	Study focus	Paradigms	Main outcomes: implementation intention effects
**Studies on cognitive processes**			
Aarts et al. (1999)	Priming	Lexical decision task, Coupon pick up	Faster responses to critical cues that mediated implementation intention effects on remembering to pick up a coupon
Webb and Sheeran (2007, 2008)	Priming (sequential)	Lexical decision task, Word-search puzzles/ Coupon pick up	Faster responses to critical cues and faster responses to response-related words when preceded by critical cues that both mediated implementation intention effects on behavior
Brandstätter et al. (2001, Studies 3 and 4)	Automaticity: efficiency and immediacy	Dual-task (Meaningless syllables or Tracking task/Go/no-go task)	Faster go-responses to critical cues in the go/no-go task without impaired meaningless syllables task performance (Study 3) or tracking task performance (Study 4)
Gollwitzer and Brandstätter (1997)	Automaticity: immediacy	Counter-argumentation task	Reduced time differences between intending to speak up during a conversation and actually speaking up
Bayer et al. (2009)	Automaticity: redundancy of conscious intent	Lexical decision task	Faster reading aloud of response-related words and faster response initiation after subliminal presentation of critical cues
Achtziger et al. (2012, Study 1)	Selective attention (auditory)	Dichotic listening task	Reduced performance on the attended headphone channel when critical cues were presented on the unattended headphone channel
Wieber and Sassenberg (2006)	Selective attention (visual)	Lexical decision/Flanker task, Letter detection task	Slower lexical decisions when words/non-words were flanked by critical cues Slow-downs were related to better letter-detection performance
Janczyk et al. (2015)	Selective attention (visual)	Psychological refractory period (PRP) task	Faster responses to Task 2 stimuli for long – but not short – stimulus onset asynchronies
Achtziger et al. (2012, Study 2)	Memory	Recall task	Improved recall of critical cues after 15 min and 2 days
Chen et al. (2015)	Meta-analysis on prospective memory	Several prospective memory tasks	Improved prospective memory performance in adults, and (sub-)clinical populations Slower responses in the ongoing task
Cohen et al. (2008)	Control of automatic processes (executive control)	Simon task, switch task	Reduced switch costs in the switch task Minimized influence of spatial location in the Simon task for the critical cue
Stewart and Payne (2008)	Control of automatic processes (stereotyping)	Shooter task Implicit association test (IAT)	Reduced automatic stereotyping, as indicated by the process dissociation procedure Faster responses in stereotype-incongruent trials and slower responses to stereotype- congruent IAT trials
Mendoza et al. (2010)	Control of automatic processes (stereotyping)	Shooter task	Reduced behavioral expression of implicit stereotypes, reduced automatic stereotype activation by distraction-inhibiting implementation intentions
Webb et al. (2012a)	Control of automatic processes (implicit attitudes)	General knowledge test, Verb identification test	Reduced unhelpful effects of primed social categories and goals
Webb et al. (2012b)	Control of automatic processes (implicit attitudes)	IAT, Go/no-go association task	Faster responses to attitude-incongruent trials in implicit attitude tests
Adriaanse et al. (2011a)	Control of automatic processes (habits)	Lexical decision task	Faster responses to non-habitual means
Wieber et al. (2014)	Control of automatic processes (social influence)	Mimicking task, spending task	Restored the positive effects of mimicking for an unlikable person Shielded individuals’ saving goal when a mimicking person challenged it
**Studies with critical samples**			
Lengfelder and Gollwitzer (2001)	Individuals with frontal lesions	Go/no-go task	Faster responses to critical cue go-trials in individuals with frontal brain lesions
McFarland and Glisky (2011)	Community-dwelling older adults	Prospective memory task	Reduced number of forgotten prospective memory responses for individuals with low as well as high frontal-lobe function (as measured by several tests including the mental arithmetic test from the Wechsler Adult Intelligence Scale – Revised)
Brom et al. (2014)	Community-dwelling older adults	Regular blood pressure monitoring, Intelligence subscale	Reduced number of forgotten blood pressure tests in participants with low fluid intelligence (i.e., poor performance digit symbol subtask from the Wechsler Adult Intelligence Scale)
Brom and Kliegel (2014)	Community-dwelling older adults	Regular blood pressure monitoring, Switch task	Reduced number of forgotten blood pressure tests in participants with limited executive abilities (i.e., high mixing costs in a switch task)
Brandstätter et al. (2001, Study 1)	Opiate addicts in withdrawal	Composing a CV in 7 h	Increased completion rates for the CV task in opiate addicts in withdrawal
Brandstätter et al. (2001, Study 2)	Individuals with schizophrenia	Go/no-go task	Faster responses to critical cue go-trials in individuals with schizophrenia
Chen et al., 2014	Individuals with schizotypal personality	Prospective memory task	Faster responses to ongoing N-back task and reduced number of forgotten key presses in response to the four prospective memory target words
Kardiasmenos et al. (2008)	Individuals with multiple sclerosis	Prospective memory task	Reduced number of forgotten prospective memory tasks during a “Virtual Week” board game
Grilli and McFarland (2011)	Memory-impaired individuals	Prospective memory task	Reduced number of forgotten prospective memory tasks during a general knowledge test in memory-impaired individuals with neurological damage
Brown et al. (2009)	Individuals with epilepsy	Medication adherence	Improved medication adherence
O’Carroll et al. (2013)	Stroke survivors	Medication adherence	Improved medication adherence
Gawrilow et al. (2011a)	Children with Attention Deficit/Hyperactivity Disorder (ADHD)	Computerized delay-of-gratification task	Improved delay of gratification
Gawrilow and Gollwitzer (2008)	Children with ADHD	Go/no-go task	Improved inhibition of unwanted responses in no-go trials, implementation intentions added to the beneficial effects of psychostimulant medication on response inhibition
Gawrilow et al. (2011b)	Children with ADHD	Modified WCST, Resistance to distractions task	Reduced executive functioning deficit, as indicated by better performance on modified Wisconsin Card Sorting Test (WCST) and on math problems with distractions
**Studies using physiological measures**			
Paul et al. (2007)	Children with ADHD	Go/no-go task, electroencephalography (EEG)	Improved response control (i.e., fewer inhibition errors following no-go trials) and increased P 300 component (i.e., no reduced no-go/go amplitude differences during the first half of the P 300 component) in children with ADHD, better inhibition correlated with higher no-go/go amplitude difference
Scholz et al. (2009)	Executive functioning under stress	Go/no-go task, cortisol level, heart rate	Improved go/no-go task under conditions of acute stress [i.e., Trierer Social Stress Test (TSST) leading to increased levels of cortisol, heart rate, and state anxiety]
Stalbovs et al. (2015)	Learning processes	Multimedia learning performance, eye-tracking	Improved multimedia learning outcomes by fostering text-picture integration (more gaze transitions between text and picture as measured by eye-tracking)
Schweiger Gallo et al. (2009)	Emotion regulation (spider fear)	Affective picture evaluation task, EEG	Reduced negative affect when viewing spider pictures and lower positivity of P100 component (i.e., down-modulation of the high positivitiy characterizing the responses to highly arousing negative stimuli) in individuals with fear of spiders
Hallam et al. (2015)	Emotion regulation (sadness and disgust)	Affective picture evaluation task, functional magnetic resonance imaging (fMRI)	Reduced negative affect when viewing disgusting and sad pictures, more activation of right inferior frontal gyrus and ventro-parietal cortex, more effective modulation of left amygdala, lower self-reported affect corresponded with relatively reduced left amygdala activity (coupling of orbitofrontal cortex and amygdala)
Gilbert et al. (2009)	Brain activity during self-initiated vs. cued responding	Prospective memory tasks, fMRI	Greater percentage of detected prospective memory cues, no increase in activity in a predominantly frontoparietal network, responses to targets yielded greater activity in rostal prefrontal cortex (medial BA 10), difference in target-related BA 10 activity mirrored differences in behavior between intentions

### Studies on Cognitive Processes

To examine the cognitive processes underlying implementation intention effects, various approaches from the literature on social-cognitive automatic processes (Bargh and Chartrand, 2000; Bargh et al., 2012) have been used: cognitive tasks such as sequential priming paradigms, dual-task paradigms, attention paradigms, and memory paradigms, as well as paradigms that focus on the control of ostensibly uncontrollable phenomena. These paradigms originated in different research traditions, and each allows the examination of a different facet of the processes underlying implementation intention effects. In the following, we review the research on priming, automaticity, selective attention, memory, and the control of automatic processes.

#### Priming

Several studies have investigated the cognitive mechanisms underlying implementation intention effects using priming paradigms (Aarts et al., 1999; Webb and Sheeran, 2007, 2008). In general, priming paradigms make use of the fact that the prior presentation of a stimulus facilitates the subsequent identification and classification of the same or a closely related target stimulus (Neely, 1991). To examine the assumed implementation intention processes, researchers have used lexical decision tasks in which participants are asked to decide whether a presented letter string represents a meaningful word or not. According to the logic of this task, faster responses to a target word (relative to neutral words) indicate the increased accessibility of the mental representation of this word.

An initial study by Aarts et al. (1999) focused on the process assumption that implementation intentions increase the accessibility of the critical cue that is specified in the if-component. Participants formed the goal to collect a food coupon on their way from the laboratory to the cafeteria and either added an implementation intention specifying when, where, and how they would pick up the coupon or not. Next, participants worked on a lexical decision task that contained five target words describing the situation specified in the plan. Subsequently, participants were sent to the cafeteria, and it was assessed whether they collected the coupon or not. Implementation intention participants responded more quickly to the target words in the lexical decision task and were significantly more successful in collecting the coupon (80%) than mere goal intention participants (50%). Importantly, a mediation analysis revealed that the effect of planning on actually collecting the coupon was no longer significant when the response times for target words were included as a covariate. As faster responses to target words indicate that implementation intentions increased the accessibility of the critical cues, this mediation suggests that the increased accessibility helped participants to translate their intentions into action.

In a second study, Webb and Sheeran (2007) also examined the second process assumption (i.e., that implementation intentions increase the strength of the cue-response link) using a sequential priming paradigm. All participants formed the goal to respond correctly and as fast as possible to words and non-words. Implementation intention participants additionally formed the plan to “press” the non-word button especially fast whenever they encountered the non-word “avenda.” Control participants familiarized themselves with this word for 30 s to keep levels of familiarity and mental rehearsal equal across conditions. Next, a lexical decision task measured cognitive processes and 24 word-search puzzles measured participants’ behavior. In the lexical decision task, a subliminally presented (i.e., for 17 ms) prime preceded each target stimulus. In line with the hypotheses, implementation intentions accelerated participants’ responses when an “avenda” prime preceded the “press” target (i.e., cue-response link) and when a neutral word preceded the “avenda” target (i.e., heightened accessibility of the critical cue). Moreover, participants with implementation intentions detected the non-word “avenda” more quickly than control participants in the subsequent word-search puzzles. Most importantly, a mediation analysis revealed that both types of acceleration contributed to the implementation intention effect on puzzle performance. These findings corroborate the assumption that both the heightened cue accessibility and the strengthened cue-response link contribute to implementation intention effects.

A third study (Webb and Sheeran, 2008) replicated and extended these effects using the Aarts et al. (1999) paradigm. This research demonstrated the simultaneous mediation of implementation intention effects by both cue accessibility and the cue-response link: again, implementation intentions helped participants to collect a coupon, and this effect was simultaneously mediated by the acceleration of their responses when critical cue primes preceded critical response targets and when neutral primes preceded critical cue targets. Overall, the findings from the reported priming studies validate the assumed roles of cue accessibility and the strength of cue-response associations in the effectiveness of implementation intentions.

#### Automaticity

In addition to these findings, another set of behavioral studies speaks to the cognitive processes underlying implementation intention effects, namely studies that have investigated whether action control by implementation intentions involves features that characterize automatic action control (Bargh, 1994; Bargh et al., 2012). Three features of automaticity have been tested: the efficiency of action control, the immediacy of response initiation, and the redundancy of a further conscious intent to act once the critical situation is encountered.

##### Efficiency

To test the assumption that action initiation by implementation intentions is efficient, Brandstätter et al. (2001, Studies 3 and 4) examined whether implementation intentions would also speed up action initiation under conditions of increased mental load using dual-task paradigms. In the elaborate paradigm of Study 4, participants simultaneously worked on a primary tracking task and a secondary go/no-go task. Both tasks involved the same modalities (i.e., visual input and motor responses) in order to ensure that they would draw on the same limited cognitive resources. Consequently, a performance improvement in the secondary task only qualifies as an increase in efficiency when it does not impair performance in the primary task. In line with this logic, implementation intention participants responded more quickly to critical stimuli in go-trials of the secondary task than control participants while performing equally well on the primary task. Similarly, a study on the consequences of forming implementation intentions observed that implementation intentions that affect goal striving by facilitating the response initiation did not impair the consideration of alternative goal-directed responses (Parks-Stamm et al., 2007, Study 2). Thus, action control by implementation intentions can be characterized as efficient.

##### Immediacy

The dual-task studies conducted by Brandstätter et al. (2001, Studies 3 and 4) also provide evidence for the immediacy of action control by implementation intentions: implementation intention participants responded faster to the critical stimulus than goal intention participants. However, the immediacy of the pre-planned response has also been tested in a more social-psychological setting using a confrontational conversation paradigm (Gollwitzer and Brandstätter, 1997). This paradigm utilizes the importance of responding immediately in social contexts by examining how long participants take to counter unacceptable remarks by others. In the study, participants watched a video of a confederate making xenophobic statements. Before they viewed this video a second time, they were asked to select scenes that they personally considered to be particularly suitable for presenting counterarguments by pressing a button and to either form implementation intentions to speak up at each of the selected scenes or not. After selecting scenes in the second run, participants’ task in the third run was to interrupt the video when a selected scene shows up and to actually comment on a statement. The time difference between the onset of the selected scenes and actually pressing the button served as an indicator of the immediacy of seizing the preselected opportunities. As predicted, implementation intention participants spoke up more quickly after the marked scenes (i.e., more immediately) than control participants. These findings provide support for the hypothesis that action control by implementation intentions is automatic in terms of the immediacy of action initiation.

##### Redundancy of conscious intent

The assumption that implementation intentions can trigger action once the critical situation is encountered without requiring further conscious intent was tested by Bayer et al. (2009). In these studies, subliminally presented implementation intention cues accelerated the reading aloud of response-related words (i.e., action preparation; Bayer et al., 2009, Study 1) and the initiation of the actual responses (i.e., action initiation; Bayer et al., 2009, Study 2). These findings corroborate the assumption that forming an implementation intention delegates’ action control to environmental stimuli, which then trigger the linked response without requiring further conscious intent.

#### Selective Attention

In addition to the research on priming and the automation of action control by implementation intentions, other studies have focused on the question of whether implementation intentions also affect attentional processes. To measure implementation intention effects on attention, behavioral paradigms focusing on auditory (Achtziger et al., 2012, Study 1), and visual selective attention (Wieber and Sassenberg, 2006) have been applied. In both paradigms, participants work on a focal task while the implementation intention cues are presented as distractors. The extent to which performance on the focal task is impaired by the presence of critical cues relative to neutral cues indicates the attraction of attention by implementation intentions.

To investigate auditory attention, participants in a dichotic listening task study (Achtziger et al., 2012, Study 1) were asked to repeat the words that were played on one channel of their headphones (i.e., the attended channel) while simultaneously ignoring words presented on the other channel (i.e., the unattended channel). The unattended channel presented cues from a self-generated implementation intention that participants had formed 1 day earlier in an ostensibly unrelated study. These cues increased latencies in repeating the words from the attended channel as well as error rates. To test visual attention, participants in a flanker task study (Wieber and Sassenberg, 2006) were asked to respond to focal cues while ignoring task-irrelevant flankers that were presented alongside. For instance, in Study 2, presenting the critical implementation intention cue “d” as task-irrelevant flankers slowed categorization responses to the focal cues, and this slowdown predicted the number of critical letters “d” participants detected in a subsequent letter detection task.

In addition, a set of experimental studies by Janczyk et al. (2015) investigated implementation intention effects on visual attention in greater detail by using a Psychological Refractory Period (PRP) paradigm (Pashler, 1994). This paradigm utilizes the established division of task processing into a pre-central perceptual stage, a central stage of response selection, and a post-central motor stage. Whereas the perceptual and motor stages are assumed to run in parallel with other processes, the central stage is not (i.e., central bottleneck assumption). In the PRP paradigm, participants simultaneously work on two tasks in each trial. Both tasks consist of distinct stimuli (S1 vs. S2) that require different responses (R1 vs. R2). The time difference between the onset of the stimuli S1 and S2 is referred to as the stimulus onset asynchrony (SOA). According to the central bottleneck assumption, short SOAs should delay responses to the secondary task (i.e., cognitive slack), as Task 2 must wait for Task 1 to pass through the central response selection stage. For long SOAs, however, no such cognitive slack should occur, as the central task-processing stages do not overlap. Assuming that implementation intentions affect the pre-central perceptual stage, no differences between implementation intentions specifying how to respond to a stimulus in Task 2 (i.e., forced-choice trials) and control task instructions (i.e., free-choice trials) should be observed, as any differences should be covered by the cognitive slack of short SOAs. For long SOAs, however, there should be no cognitive slack to cover differences at the perceptual stage, and thus the implementation intention effects on perception should translate into faster responses in Task 2. In line with this reasoning, implementation intention participants responded faster than control participants to Task 2 stimuli for long SOAs but not for short SOAs.

In sum, evidence from behavioral paradigms focusing on both auditory and visual selective attention indicates that implementation intentions affect perceptual and attentional processes. This is in accordance with the assumption that implementation intentions facilitate the detection of the specified situational cues. The fact that these effects took the form of diminished performance in the dichotic listening task (Achtziger et al., 2012, Study 1) and the flanker task (Wieber and Sassenberg, 2006) also points to the uncontrollability of these effects, which is another feature characterizing automatic processes (Bargh, 1994; Bargh et al., 2012).

#### Memory

Implementation intention effects on memory have also been examined. For instance, (Achtziger et al., 2012, Study 2) tested the assumption that forming implementation intentions improves the recognition of anticipated situations using a recall paradigm. They observed better recall of implementation intention cues relative to the recall of non-specified cues both 15 min and 2 days after the formation of the if-then plan. In addition to these memory-enhancing effects, research on prospective memory has examined whether implementation intentions can help overcome the gap between intention formation and delayed enactment. Prospective memory refers to remembering to perform a planned action at a future point in time (Einstein and McDaniel, 1990; McDaniel and Einstein, 2000; Brandimonte et al., 2014). From a prospective memory perspective, forming implementation intentions thus qualifies as a specific method of intention encoding. Beyond these conceptual similarities, there is also an overlap with regard to the paradigms that have been employed to examine the effects of implementation intentions and prospective memory. Similar to the dual-task paradigms used to investigate the efficiency of implementation intentions, typical prospective memory paradigms combine an ongoing primary task with a secondary task. However, whereas the secondary tasks in the research on efficiency require continuous responses, the secondary tasks in prospective memory research require responses to prospective memory stimuli that are presented only sporadically. For example, participants perform a working memory task that asks them to recall the last three words presented on the screen whenever the word RECALL appears, and, as a second task, they are asked to write the day of the week in the upper right-hand corner of each sheet of paper they receive (Chasteen et al., 2001). Relative to regular encoding methods that do not employ the if-then format, implementation intentions improved prospective memory performance with a medium effect size of *d* = 0.45 (meta-analysis of 36 studies by Chen et al., 2015). With regard to the processes involved, implementation intentions slowed down responses in the ongoing task though the effect size was small (*d* = 0.22). Although the source of these slowdowns has not yet been clarified, these costs of implementation intentions for the ongoing task suggest that implementation intention effects on prospective memory are not solely automatic but involve a complex interaction of automatic and controlled processes (see Bugg et al., 2013; Burkard et al., 2014; Chen et al., 2015).

#### Control of Automatic Processes

A final set of behavioral studies provides insights into the automaticity of implementation intention effects by examining whether they can control phenomena that are known to be automatic. These automatic phenomena are usually not amenable to deliberate intentional control; successfully controlling them would thus indicate that action control by implementation intentions is automatic. In accordance with the horse-race metaphor (Dunbar and MacLeod, 1984), implementation intentions might outrun competing automatic processes. In an application of this reasoning, implementation intentions have been examined in various cognitive and social-psychological paradigms that are characterized by their high stability (i.e., the effects are difficult to change). However, because such modifications of automated processes do not seem promising as a starting point for physiological research on implementation intentions, we will only briefly review these findings. In support of the automaticity of action control by implementation intentions, they improved cognitive control performance in Simon tasks and in task switching paradigms (Cohen et al., 2008); they allowed participants to control automatic stereotype activation (Stewart and Payne, 2008), stereotype application (Mendoza et al., 2010), effects of primed social categories and goals (Webb et al., 2012a), and implicit attitudes (Webb et al., 2012b); and they enabled participants to break unwanted snacking habits (Adriaanse et al., 2011a) and to resist unwanted social influences (i.e., effects of being mimicked; Wieber et al., 2014).

#### Summary

We reviewed a variety of behavioral approaches that sought to provide insights into the cognitive processes that characterize action control by implementation intentions. In support of implementation intention theory, implementation intentions have been found to facilitate the detection of critical cues as well as the initiation of the associated responses after the respective cues. Moreover, implementation intention effects have been shown to be automatic in terms of their efficiency, immediacy, and the redundancy of further conscious intent. Finally, implementation intentions have been demonstrated to affect both attentional and memory processes and to be capable of controlling automatic processes. Behavioral studies have thus provided multi-faceted insights into the cognitive processes underlying implementation intention effects. However, implementation intention theory can also be informed by behavioral studies in another way – namely, by taking into account implementation intention studies with critical samples.

### Studies with Critical Samples

To explore the prerequisites of implementation intention effects and to determine who can benefit from forming implementation intentions, studies have been conducted with critical samples that are known to experience difficulties with self-regulating their actions. In support of the assumption that implementation intentions automate action control, a recent meta-analysis of implementation intention effects in individuals with mental-health problems (Toli et al., in press) found that implementation intentions had a large-sized effect on goal attainment (*d*_+_ = 0.99). In 29 experimental studies with 1,636 participants, implementation intentions proved effective across different mental-health problems and goals. In the following section, we discuss selected studies from this meta-analysis, including individuals with frontal-lobe lesions, opiate addicts experiencing withdrawal, individuals with schizophrenia, and children with Attention Deficit/Hyperactivity Disorder (ADHD), but also other studies with critical samples that can inform physiological research on implementation intentions, such as older adults with limited executive abilities, individuals with multiple sclerosis, memory-impaired individuals with neurological damage, and individuals suffering from epilepsy or the effects of strokes.

#### Adult Samples with Reduced Frontal-Lobe Function

One of the brain areas that have most consistently been associated with action control is the prefrontal cortex (reviews by Shallice, 1988; Passingham, 1993; Miller and Cohen, 2001; Alvarez and Emory, 2006; Heatherton and Wagner, 2011; Stuss and Knight, 2013; Casey, 2015). Corroborating this assumption, Lengfelder and Gollwitzer (2001, Study 1) found that participants with frontal-lobe lesions performed worse on a deliberation task (i.e., weighing the pros and cons of different decision alternatives) than participants with non-frontal-lobe injuries and control participants. In a further experiment (Lengfelder and Gollwitzer, 2001, Study 2), the researchers argued that self-regulation through implementation intentions should require less activity in frontal areas than deliberate action control by mere goals and should therefore support action control in individuals with brain-injuries. In line with this reasoning, frontal-lobe patients, non-frontal-lobe patients, and student controls all responded more quickly in the secondary task (go/no-go task) of a dual task paradigm when encountering a critical target (i.e., the number 3) that was specified in an implementation intention. Indeed, patients with a low performance on the Tower of Hanoi problem-solving task, a common measure of the capability for deliberate action control, profited the most from forming implementation intentions.

Further support for the assumption that implementation intentions do not require high frontal-lobe function to improve goal attainment comes from studies involving community-dwelling older adults (meta-analysis by Chen et al., 2015). McFarland and Glisky (2011) observed that older adults with high and low frontal-lobe function profited alike from forming implementation intentions for an event-based prospective memory task. Brom and Kliegel (2014) and Brom et al. (2014) also found that implementation intentions helped community-dwelling older adults to remember their intention to measure their blood pressure regularly throughout the day. However, they observed that these implementation intention effects were moderated by participants’ cognitive abilities (fluid mechanics; Brom et al., 2014) and executive control (task switching; Brom and Kliegel, 2014), such that implementation intentions particularly helped participants with low rather than high fluid intelligence and task switching abilities. Interestingly, the study conducted by Brom and Kliegel (2014) compared the impact of two different interventions on prospective memory tasks. In addition to an implementation intention condition that was intended to reduce the executive demand posed by the prospective memory task, another intervention was designed to train the executive functions needed for prospective memory (i.e., task switching). Their results indicate that task-switching training was effective at improving this particular executive function, but that this effect did not generalize to the mundane setting of measuring one’s blood pressure. Implementation intentions, on the other hand, improved adherence with the blood-pressure measurement schedule, and follow-up analyses showed that this improvement was most notable in those participants who were most challenged by the prospective memory task (i.e., those with limited task-switching abilities).

#### Adult Samples with Neurological Conditions

In addition to reduced frontal-lobe function, other clinical and subclinical conditions are also known to be associated with self-regulation problems and should thus benefit from the effects of implementation intentions. For example, in a study by Brandstätter et al. (2001, Study 1), opiate addicts experiencing withdrawal were asked to write a CV for a new job application before the end of the day. Half of the participants furnished this goal with an implementation intention (i.e., they planned out when and where to write the CV); the other half (control condition) formed irrelevant implementation intentions (when and where to eat lunch). Although all patients were highly motivated to write the CV, no patient in the control condition finished it in time. However, 80% of the implementation intention participants succeeded in doing so. Implementation intentions thus helped opiate addicts in a state of withdrawal to translate their intentions into action despite their presumed preoccupation with their cravings (i.e., high cognitive load). Similar to this example, other critical samples were also found to benefit from planning with implementation intentions: Schizophrenic patients responded faster to critical numbers in a go/no-go task (Brandstätter et al., 2001, Study 2) and young adults with schizotypal personality features performed better on a prospective memory task (Chen et al., 2014). Patients with multiple sclerosis (Kardiasmenos et al., 2008) as well as memory-impaired individuals with neurological damage (Grilli and McFarland, 2011) improved their prospective memory performance, and individuals with epilepsy (Brown et al., 2009) and stroke survivors (O’Carroll et al., 2013) increased their adherence to their medication schedules.

#### Samples of Children with ADHD

In addition to both younger and older adults, the effectiveness of implementation intentions in critical samples has also been tested in children, specifically in children suffering from ADHD. Children with ADHD find it difficult to control their impulses and to delay immediate gratification (Rodriguez et al., 1989). In a study, Gawrilow et al. (2011a, Study 1) asked children diagnosed with Hyperkinetic Disorder (F90.0, ICD-10; WHO, 1991) to play a computerized delay-of-gratification task in which they could earn additional money by delaying their response. Each of the 40 task trials started with a red picture showing an animal or a transport vehicle before a blue picture showing an animal or a transport vehicle followed after 30, 40, 50, or 60 s. Children could skip waiting for the blue picture by pressing a button at any time. However, whereas they earned only one point (worth 5 ct) when clicking in response to red pictures, they earned three points (worth 15 ct) when clicking in response to blue pictures. Children were randomly assigned to one of three experimental conditions. One-third of the children received only the task instruction that red pictures are worth one point and blue pictures are worth three points. One-third of the children added the goal intention “I will earn as many points as possible,” to this task instruction, and one-third formed the goal intention and added the implementation intention “Whenever a red picture appears, then I will wait for the blue one.” Children then performed the task. In line with previous research on ADHD and delay of gratification, children in the control condition were not very successful at waiting for the blue picture, earning 3.35€; out of a possible 6€ on average. Speaking to the limited power of goals to increase the delay of gratification, children who had formed the goal intention did not significantly differ from the control group (they earned on average 2.82€ out of 6€). However, children who had formed the implementation intention managed to delay gratification much longer, earning close to the maximum 6€ possible (5.54€ on average). Thus, even those most prone to impulsive behavior managed to curb their impulses and delay gratification by forming implementation intentions. Similarly, Gawrilow et al. (2011b) also found that implementation intentions benefit executive functions (i.e., shifting, resistance to distraction) in children with ADHD.

#### Summary

Studies with critical samples have provided converging evidence that implementation intentions can be successfully formed, maintained, and executed by people suffering from impaired self-regulation. Implementation intention effects were observed under conditions of reduced frontal-lobe functionality, opiate withdrawal, schizophrenia, epilepsy, prior strokes, multiple sclerosis, neurological damage with memory impairment, and ADHD. These findings from behavioral studies in critical samples are also in accordance with the proposition that implementation intentions automate action control by shifting from top–down to bottom–up information processing. Self-regulation with implementation intentions thus depends less heavily on frontal-lobe processes and indeed requires fewer cognitive resources than self-regulation with mere goals.

### Studies with Physiological Measures

Although the findings from studies on cognitive processes and those involving critical samples provide cumulative evidence of the assumed implementation intention effects and processes, the paradigms used in these studies offer only limited insights into how a certain response or behavior comes about. Physiological measures such as heart rate and cortisol level, analyses of eye movements, and electrocortical and imaging measures open new venues for the investigation of implementation intention effects. Neuroscientists have begun to explore what implementation intention research can learn by using these physiological measures, either as a central outcome measure or as a complementary measure.

#### Cortisol and Heart Rate Studies

To examine whether implementation intentions improve cognitive performance even under conditions of acute stress, Scholz et al. (2009) manipulated participants’ stress levels and their intentions regarding a subsequent go/no-go task. First, they either formed an implementation intention or received standard instructions on how to perform an upcoming go/no-go task. Next, they either engaged in the Trier Social Stress Test (Kirschbaum et al., 1993), which required them to give an improvised speech that was videotaped, or they rested (control condition). Finally, all participants worked on the go/no-go task. To ensure that participants were indeed experiencing acute stress, their cortisol levels and heart rates were measured after the stress manipulation. Implementation intention and goal intention participants exhibited equally elevated cortisol responses and heart rates in response to the stress induction; consequently, self-regulation capabilities should be equally impaired in the two conditions. The responses in the go/no-go task showed that stress induction led to worse performance in comparison to the control condition. However, an interaction indicated that this effect only occurred in the goal intention condition; implementation intention participants’ performance was unaffected by the stress induction. This study exemplifies how implementation intention research can apply physiological measures, not only as primary outcome measures but also as complementary measures to investigate how different physiological states impact the effectiveness of implementation intentions.

#### Eye-Tracking Studies

A study by Stalbovs et al. (2015) is another good example of how the measurement of physiological processes can contribute to implementation intention research. The authors used eye-tracking data to gain insight into how implementation intentions facilitate multimedia learning. They observed that learners who formed implementation intentions to integrate the information presented in text paragraphs and picture elements looked back and forth between the two representational formats more frequently, and that these transitions predicted the subsequent learning outcomes. This study also demonstrates how measuring eye movements can complement behavioral measures in order to increase our understanding of the processes underlying implementation intention effects.

#### Electrocortical Studies

High temporal resolution is required to investigate the temporal dynamics of implementation intention effects and to shed light on the processes that take place before individuals initiate their responses. Electroencephalography (EEG) records the electrical activity along the scalp non-invasively and with a high temporal resolution using electrodes. One challenge of EEG measures is the differentiation between a response of interest (e.g., the emotional response to seeing a picture) and various other processes taking place at the same time (e.g., thinking of one’s vacation, wondering what the study is about). In order to investigate the response to a particular event (e.g., the emotional picture), EEG researchers often rely on event-related potentials (ERPs). The main idea of ERPs is to repeatedly create an event (e.g., repeatedly viewing the emotional picture), record each electrical response, and then calculate an average across all instances. Thereby, the other processes that occur at random should average out across measurements, rendering the “substrate” response to the event of interest observable (Luck, 2014). The high temporal resolution of such ERP measures allows the observation of cortical responses that occur much earlier than behavioral responses (e.g., button pressing) in the cognitive paradigms discussed above. Because strategic response control by goal intentions needs “attentional (effortful) resources that take time to accrue” (Bargh and Chartrand, 2000, p. 273), behavioral response time approaches only directly measure effects that occur at least 300 ms after the stimulus onset. Thus, although response time approaches can indirectly capture earlier processes (e.g., using the PRP paradigm), only ERP measures can measure effects occurring immediately after the onset of a stimulus; they can therefore put the immediacy hypothesis of implementation intention theory to a critical test.

##### The P300 ERP component

One of the ERP components is the P300 in tasks measuring inhibitory control (endogenous evaluation of response control and conflict monitoring) such as go/no-go tasks. In go/no-go tasks, some types of stimuli (e.g., numbers) require a response, whereas others (e.g., letters) do not. No-go stimuli have been found to evoke higher P300 amplitudes than go stimuli. The effort to override the impulse to respond is thus reflected in an elevated P300 response. This amplitude increase in response to no-go stimuli is less pronounced in children with ADHD (Fallgatter et al., 2004), which is in line with the observation that children with ADHD experience difficulties with inhibiting their responses. Building on these findings, several studies have addressed the question of whether implementation intentions can be used to improve response inhibition performance and to increase the P300 response. Behavioral data shows that implementation intentions indeed improve no-go performance in children with ADHD (Gawrilow and Gollwitzer, 2008, Studies 1 and 2). Paul et al. (2007) investigated whether this behavioral change is also reflected in ERP data. Indeed, replicating the earlier findings, if-then plans improved response inhibition, and this improvement was also reflected in the ERPs: implementation intentions increased the differences between go and no-go trials in the first half of the P300 (between 160 and 312 ms) in children with ADHD to such an extent that they did no longer differ from control children. As discussed above, this early activation validates the strategic automaticity created by implementation intentions and extends the response inhibition findings from the behavioral studies. In the second half of the P300 (312–452 ms), however, implementation intentions reduced the amplitude in control participants, but the amplitude remained high in participants with ADHD. This finding suggests that implementation intentions reduced the processing effort in children without ADHD but not in children with ADHD. It may be the case that implementation intentions helped ADHD children to recognize the specified situation (no-go signal), but that the inhibition of the impulse to respond was still effortful. In line with this interpretation, the response times indicated that ADHD children responded more slowly than control children, especially in the planning condition. This interpretation is also in accordance with behavioral studies that have demonstrated that implementation intentions can improve the initiation of reflective responses such as evaluating a chosen strategy (Henderson et al., 2007), taking an onlooker’s perspective (Wieber et al., 2015), and reviewing the pros and cons of different decision alternatives (Thürmer et al., 2015b). Overall, the study by Paul et al. (2007) shows that implementation intentions can affect neural activity, as reflected in the first half of the P300 component, and improve response inhibition in children with ADHD.

##### The P100 ERP component

Another well-studied ERP component is the P100 in emotion processing, a factor commonly interpreted as an indicator of early visual perception that is beyond strategic control by goal intentions (Rellecke et al., 2012). Schweiger Gallo et al. (2009, Study 3; overview by Schweiger Gallo et al., 2013) used such ERP measures to contextualize self-report data. This self-report data indicated that the implementation intention “And if I see a spider, then I will ignore it!” enabled participants with chronic fear of spiders to better control their fear in response to spider pictures than either the mere goal intention “I will not get frightened” or viewing the pictures without further instructions. According to the ERP measures, only participants who bolstered their goal intention to not get frightened with an ignore-implementation intention exhibited significantly reduced early activity in the visual cortex in response to spider pictures, as reflected in a smaller occipital P100 (assessed at 120 ms after a spider picture was presented). This modification of early perceptual processes provides further evidence of the strategic automation of the specified goal-directed response (here, an ignore response) when the critical cue (here, a spider picture) is encountered. Alternative explanations including differences in ocular movements, commitment, and self-reported success at not getting frightened could not account for these findings. The observed effects are therefore in line with the prediction that implementation intentions allow more automatic emotion regulation.

#### Imaging Studies

Although EEG research indicates that implementation intentions can help control very early responses, the notion of strategic automaticity also has implications with regard to the brain areas involved. Because EEG only provides very limited insights into the localization of neural activity, imaging techniques represent a further promising approach for the investigation of the neural correlates of implementation intention effects. The strategic automation of action control by implementation intentions should also be reflected in the location of neural activity: It should draw more on brain areas that are associated with stimulus-guided, automatic bottom–up processing. To examine the origins of implementation intention effects, the high spatial resolution of functional magnetic resonance imaging (fMRI) offers a useful approach. Using fMRI allows deriving a correlate of the blood oxygen level in the brain (blood-oxygen-level dependent, BOLD), which is usually interpreted as a sign of activity (i.e., higher levels of activity are reflected in more oxygen in the blood; e.g., Aguirre et al., 1998). Because fMRI is commonly combined with high-resolution structural MRI scans, one can locate this activity quite accurately. Recent fMRI research indicates that different kinds of action control use different brain regions. In general, while bottom–up, stimulus-driven, or automatic action control is linked to more medial regions (e.g., the orbitofrontal cortex, OFC or the medial rostral prefrontal gyrus, medial Brodmann Area 10, BA 10), top–down, deliberate control of response initiation seems to employ more lateral areas (such as the dorsolateral prefrontal gyrus, DLPFC, or the lateral BA 10; meta-analyses by Gilbert et al., 2006; Diekhof et al., 2011; Buhle et al., 2014). The observation that implementation intentions support stimulus-driven, automatic action control therefore suggests that medial regions in the frontal cortex should be more active during action control with implementation intentions, and that lateral regions should be more active during deliberate action control with mere goal intentions.

##### BOLD activity during emotion regulation

This idea has recently been tested in the context of emotion regulation by Hallam et al. (2015). As discussed above, implementation intentions have been shown to help regulate emotions more effectively and efficiently (i.e., modulating even early perceptual responses). A recent fMRI study tested whether this emotion regulation by implementation intentions indeed originates in the medial prefrontal areas that have been implicated in automatic emotion control (Etkin et al., 2011). While in the scanner, participants viewed a number of disgusting, sad, and neutral pictures. Before each of the emotional picture trials, participants were either asked to merely view the picture (“attend”) or were prompted to use one of two strategies to regulate their emotional response: Adopting a detached and unemotional attitude (“reappraise”) or attempting not to get emotional (“suppress”). Implementation intention participants had learned about these two strategies in an if-then format (“If I see REAPPRAISE, then I will tell myself that ‘these are just pixels on a screen and the picture can’t get to me!’ ” and “If I see SUPPRESS, then I will block out all bad feelings and just stay cool!”); control participants merely formed the respective goal intentions (“When viewing pictures preceded by REAPPRAISE you should adopt a detached and unemotional attitude” and “Some picture will be preceded by the word SUPPRESS. When viewing these pictures you should try to stop yourself from getting emotional. In other words, try to suppress any feelings you have when looking at the picture”). The results showed that, as expected, implementation intentions employed the OFC, while goal intentions activated the more lateral DLPFC and regions outside the prefrontal cortex (Temporoparietal Junction, TPJ). Going one step further, connectivity analyses demonstrated that this implementation intention effect resulted in lower amygdala BOLD activity after participants viewed emotional pictures, a finding that was in line with their self-reports (i.e., participants reported less emotional responses in implementation intention trials). These new data therefore tie in nicely with the behavioral (Webb et al., 2012c) and electrocortical (Schweiger Gallo et al., 2009) findings on emotion regulation using implementation intentions discussed above.

##### BOLD activity during prospective memory tasks

Beyond emotion regulation, these differences between mere goals and goals with implementation intentions should also be reflected in the brain areas active during prospective memory for action control. In particular, the BA 10 should be involved: Although this medial prefrontal region is not yet well understood and serves multiple functions (Amodio and Frith, 2006; Gilbert et al., 2006; Van Overwalle, 2009; Denny et al., 2012), brain activity in the medial BA 10 seems to be associated with bottom–up (stimulus-initiated) prospective action, and brain activity in the lateral BA 10 seems to be related to top–down (self-initiated) prospective action (Burgess et al., 2007). Therefore, if implementation intentions actually delegate action control to external stimuli, the medial BA 10 should be involved in their execution, and if goal intentions actually require deliberately monitoring of the environment, they should employ the lateral BA 10. Gilbert et al. (2009) tested this hypothesis in an fMRI study in which participants had to perform a prospective memory task on the basis of either goal or implementation intention instructions. Acting on the basis of goal intentions was associated with brain activity in the lateral rostral prefrontal cortex (lateral BA 10), whereas acting on the basis of implementation intentions was associated with brain activity in the medial rostral prefrontal cortex (medial BA 10). Interestingly, although implementation intention participants performed better in the prospective memory task, overall they showed reduced brain activation during task performance. This finding suggests that action initiation by implementation intentions is indeed less resource-intensive than action control by mere goal intentions (e.g., Bayer et al., 2009), even in terms of the neural processes involved. This interpretation also corresponds to the results of the EEG study discussed above, in which implementation intentions improved go/no-go performance while reducing brain activity in control children (Paul et al., 2007). Given the continuing advances in research on the functions of the BA 10, future studies have a high potential to inform implementation intention research and to inspire new hypotheses on how if-then planning works. This exemplifies how physiological studies can improve our understanding of intentional action control and foster the conceptual and empirical integration of research including the neural, cognitive, and behavioral level.

#### Summary

We reviewed behavioral studies that applied physiological measures to investigate the limits, processes, and effects of implementation intentions. High cortisol levels and increased heart rates indicated the successful induction of acute stress, which did not compromise implementation intention effects. Eye-tracking allowed quantification of the enactment of the visual action strategies (i.e., gaze transitions) that were specified in implementation intentions and their mediating role with regard to the behavioral effects (i.e., multimedia learning). Electrocortical studies showed that implementation intentions affect information processing as early as 120 ms (i.e., P100 activity) and 160–312 ms (i.e., first half of the P300 activity) after the stimulus onset. Finally, imaging studies observed that action control by implementation intentions involves more activity in brain regions that are associated with bottom–up rather than top–down information processing. Thus, although only a limited number of implementation intention studies have applied physiological measures, the reviewed studies provide complementary support for the assumptions of implementation intention theory and new insights into the timing and location of implementation intention effects.

## Discussion

In this review, we examined behavioral and physiological studies that offer insights into how implementation intentions affect the translation of individuals’ intentions into action. We started by reviewing research that documented the pervasiveness of the intention–behavior gap. We laid out the conceptual framework of implementation intention theory and the processes that have been postulated in research on implementation intentions. Next, we reviewed studies on cognitive processes, studies involving critical samples, and studies assessing physiological measures.

### Studies on Cognitive Processes

The studies on cognitive processes employed a broad variety of paradigms to examine specific aspects of the strategic automation of action control by implementation intentions. Findings from priming paradigms provide support for the process assumptions of implementation intention theory, namely that if-then planning increases the accessibility of the critical cue and strengthens the link between the mental representations of the cue and response. Data from dual-task paradigms and priming paradigms show that action control by implementation intentions is characterized by features of automaticity; the response initiation is efficient, immediate, and no further conscious intent is required once the critical situation is encountered. Moreover, findings from attention paradigms such as the dichotic listening task, flanker task, and PRP paradigms demonstrate that implementation intentions affect even early perceptual and attentional processes and cannot be easily controlled. Prospective memory paradigms have also been employed to investigate implementation intention effects. These studies have found that implementation intentions can improve prospective memory; however, this improvement comes at the cost of performance in secondary tasks, suggesting that action control by implementation intentions can automatically initiate responses but that their execution may still rely on controlled processes. Finally, behavioral studies also observed that implementation intentions can be used to control automatic effects, as measured in priming paradigms, implicit attitude tests, and behavioral measures. Although the overall findings on the cognitive processes underlying implementation intentions are fairly consistent, they also indicate potential venues for future research. For example, the concepts that have been used to understand the cognitive processes involved (i.e., priming, automaticity, selective attention, and memory) originate from different research traditions (i.e., social psychological and cognitive science); accordingly, the conceptual and empirical overlap between these fields should be further promoted. Moreover, future research could follow up on the reported findings by examining the moderators of the automaticity of implementation intention effects on prospective memory as well as on other performance domains.

### Studies with Critical Samples

Implementation intention effects were also found in critical samples that suffer from impaired self-regulation. The investigated samples included individuals with reduced frontal-lobe functions, schizophrenia, epilepsy, a history of stroke, multiple sclerosis, memory impairment, and neurological damage, as well as opiate addicts and children with ADHD. The findings are consistent with the proposition that implementation intentions automate action control by shifting from top–down to bottom–up information processing as outlined in implementation intention theory. However, the studies on implementation intention effects on prospective memory in community-dwelling older adults were not conclusive with regard to the role of individuals’ executive functions. Whereas one study observed implementation intention effects for older adults with high executive functions (McFarland and Glisky, 2011), three studies (Lengfelder and Gollwitzer, 2001; Brom and Kliegel, 2014; Brom et al., 2014) observed the strongest implementation intention effects for older adults with limited executive function. Future research could systematically explore the variables that moderate implementation intention effects for older adults with high executive function in greater detail. For example, depending on the task there might be ceiling effects that prevent implementation intentions from further improving individuals’ prospective memory performance. With respect to the behavioral paradigms that have been applied in these studies, the studies exhibited substantial overlap with the behavioral studies on the cognitive processes of implementation intention effects. However, they also applied specific measures to assess individuals’ executive functions in the critical samples (e.g., frontal-lobe function, fluid mechanics, switching abilities, and response inhibition). Such measures may also serve to complement behavioral studies with physiological data, thus integrating the different areas of research on implementation intentions.

### Studies using Physiological Measures

Whereas a large number of behavioral studies have examined the cognitive processes of implementation intention effects and critical samples, only a few have used physiological approaches. These latter studies point to the usefulness of such complementary approaches. The physiological measures implemented allowed precise exploration of the contextual limits and processes: by using physiological measures including cortisol levels, heart rates, and eye movements, studies demonstrated that acute stress does not impede implementation intention effects on performance and that implementation intentions referring to visual attention indeed improved multimedia learning performance by affecting individuals’ eye movements. Moreover, electrocortical and imaging studies allowed exploration of the timing and location of implementation intention effects. EEG studies observed that implementation intentions definitely affect early information processing, as indicated by changes in P100 and P300 activity. Using fMRI, studies also showed that implementation intentions affect the spatial location of action-control processes. Whereas emotion regulation with implementation intentions was more strongly associated with the activation of brain areas previously reported to be involved in automatic emotion regulation, emotion regulation with goal intentions was associated with those previously reported to be involved in effortful emotion regulation. With regard to implementation intention processes, improved cue detection may be reflected in the increased activation of the right inferior frontal gyrus and ventroparietal cortex, and the cue-response link may be reflected in the more effective modulation of the left amygdala, as well as the coupling of the OFC and the amygdala. In contrast, emotion regulation with goal intentions resulted in increased activity in the more dorsolateral prefrontal cortex and regions outside the prefrontal cortex (TPJ), as well as more amygdala activity in response to viewing emotional pictures.

The observation that action control by implementation intentions involves more activity in the medial rostral prefrontal cortex and less in the lateral rostral prefrontal cortex is in line with the strategic automation assumption, as it suggests that action control by implementation intentions is indeed less effortful than action control by goal intentions. Building on the finding that implementation intentions affect emotion regulation processes as well as behavior regulation, various directions seem interesting for future research. For example, the physiological correlates of different types of implementation intentions (such as task-facilitating versus distraction-inhibiting implementation intentions) could be explored. Additionally, heart rate and cortisol measurements and eye-tracking could be utilized not only as complementary measures but also as direct outcome measures. Further research could investigate whether action control by implementation intentions can be used to regulate heart rate and cortisol levels. For instance, implementation intentions addressing individuals’ emotion regulation could be applied to alter effort-related physiological adjustments during task performance. In general, motivation has been demonstrated to be systematically linked to cardiovascular responses (Wright and Gendolla, 2012). Motivation effects are influenced by mood states, such that sadness primes processed during task performance lead to stronger cardiovascular responses than happiness or anger primes (Gendolla, 2012). Thus, implementation intentions directed at reducing negative affect or at eliciting positive affect during task performance should reduce these mood-induced changes in cardiovascular responses (e.g., heart rate, cardiac pre-ejection period, or systolic blood pressure). Similarly, implementation intentions that focus on effective stress management (e.g., Storch et al., 2007) should result in reduced cortisol levels.

### Methodological Considerations

Overall, the results of the studies on cognitive processes, those involving critical samples, and those implementing physiological measures provide convergent support for the process assumptions of implementation intention theory. These findings also offer first illustrations of how these methods complement each other; they address our research question of how implementation intention effects arise more effectively and comprehensively than either method on its own. Depending on the research question, studies have applied either multiple behavioral or physiological measures or a combination of the two. For instance, Aarts et al. (1999) measured the accessibility of the mental representation of the cue specified in the if-part (i.e., cognitive process measure) and subsequently observed whether participants remembered to collect a coupon (i.e., behavior). Schweiger Gallo et al. (2009, Study 3) combined self-report measures of emotions with ERP measures in order to examine how neurophysiological activity relates to participants’ subjective emotional experiences. Hallam et al. (2015) went one step further, analyzing how different brain areas impact one another: they found that implementation intention effects originated in the OFC and resulted in reduced amygdala activity. Again, this change in brain activity was reflected in participants’ self-reported emotions. These researchers also used skin conductance response measures to analyze physiological responses to the emotional stimuli. Interestingly, the neural and self-report changes in emotion were not reflected in skin conductance response changes (i.e., no differences in skin conductance between conditions were observed). This observation is well in line with the common finding that different measures of emotions are not always coherent and therefore cannot replace each other (e.g., Russell, 2003; Mauss et al., 2005; reviews by Mauss and Robinson, 2009; Reisenzein et al., 2013), which further supports our argument that a multi-method approach to the intentional control of action is necessary. Such a multi-method approach would also allow the investigation of the assumption that implementation intention effects are dependent on the activation of the superordinate goal (Gollwitzer, 1999). Although initial support for this assumption has been found in behavioral studies (Sheeran et al., 2005), combining behavioral measures with ERP or fMRI measures would provide a more differentiated test of this moderation effect. Given the broad variety of behavioral and physiological measures that implementation intention research has already successfully applied, we are confident that future studies on implementation intention processes and effects can easily tailor their methods to test their specific research hypothesis and further advance this vibrant field of research.

Moreover, different brain-imaging techniques may be combined to analyze source-effect relations (e.g., EEG and fMRI data; Ullsperger and Debener, 2010), and methodological advances in the ambulatory measurement of brain/body imaging might also permit researchers to synchronously record participants’ motor behavior, brain activity, and other physiological aspects, as well as their physical environment and external events (research topic by Gramann et al., 2014). Although the joint multistream analysis of recorded mobile brain/body imaging data poses major conceptual, mathematical, and data-processing challenges, this development would allow a more comprehensive integration of the different methods as well as a more complex examination of (social-)psychological phenomena such as joint attention (Wykowska et al., 2014) and intentional action control during negotiations (Trötschel and Gollwitzer, 2007), thereby making substantial contributions to implementation intention research.

In addition to the combination of different measures, new methods can be applied to address questions on the effectiveness of implementation intentions. For example, computational modeling (e.g., Hübner et al., 2010) and advanced mouse-tracking analytic techniques (Hehman et al., 2014) represent promising opportunities to advance our understanding of implementation intention processes and to link cognitive processes and behavior. Moreover, steady-state visual evoked potentials (ssVEPs) may be used to trace how visual stimuli are processed (Keil et al., 2003; Perlstein et al., 2003). As this method can simultaneously observe the processing of two competing stimuli, it might permit analysis of the perceptual processes involved in the link between the if-cues and the then-responses of an implementation intention in sequential priming paradigms (e.g., Webb and Sheeran, 2007).

### Conceptual Considerations

The findings on the physiological processes underlying implementation intention effects might also be used to refine the concepts used in implementation intention research. In fact, various efforts are currently underway. For example, Martiny-Huenger et al. (in press) have suggested an embodied cognition perspective to further our understanding of how implementation intention effects come about. They argue that forming an if-then plan leads to more efficient action when the activity patterns during planning resemble the activation patterns during plan execution (pattern-overlap principle). This perspective links research on implementation intentions with research on motor processes. This might be especially interesting, as rules and task sets often take the form of if-then plans. Thus, implementation intentions could potentially contribute to speed-up effects in rule conditions relative to no-rule conditions (e.g., Randerath et al., 2013, 2014).

Similar efforts to foster the conceptual and empirical integration of cognitive and physiological approaches are ongoing in other areas as well, including cognition–motivation interactions (e.g., Braver et al., 2014), prospective memory (Cona et al., 2015b), the understanding of intentions (e.g., Chiavarino et al., 2012), group processes and intergroup relations (Forbes, 2014), and priming effects (e.g., Lerner and Shriki, 2014; Tartaglia et al., 2015). These approaches may also be helpful in deriving hypotheses on how implementation intentions affect neurophysiological processes and in further integrating the different research areas. For example, given the control of priming effects by implementation intentions, findings on the neurophysiological correlates of priming effects that have been identified using EEG (Holcomb, 1988; Holcomb and Neville, 1990; Swick, 1998; Rossell et al., 2003; Franklin et al., 2007; Smith et al., 2007; Du et al., 2014; van Vliet et al., 2014) and fMRI measures (Henson, 2003; Henson and Rugg, 2003; Rossell et al., 2003) could be used to derive further hypotheses regarding the neurophysiological correlates of implementation intention effects.

### Implications for Research on the Formation and Maintenance of Implementation Intentions

Finally, the present review only examined the execution of implementation intentions. In addition to investigating the consequences of having formed implementation intentions, future research might also examine the processes occurring during the formation and maintenance of implementation intentions (see Cona et al., 2015a). For example, the formation of different kinds of intention formation strategies has been assessed using continuous magnetoencephalographic (MEG) activity (Achtziger et al., 2009). In their study, Achtziger et al. (2009) examined the self-regulation strategy to mentally contrast one’s desired positive future with the obstacles in one’s present reality (Oettingen et al., 2001). This self-regulation strategy has been found to foster the formation of strong goal intentions and to sucessfully improve goal attainment (overviews by Oettingen, 2012; Oettingen et al., 2013). As mental contrasting should be cognitively more demanding than indulging in mindless daydreaming, increased activity in prefrontal, frontal, parietal, and temporal areas was expected and found. These activity patterns indicate that mental contrasting does indeed involve strong intention formation, working memory, and episodic memory. Moreover, the heightened activity of occipital areas during mental contrasting compared to resting and indulging suggests that mental contrasting involves the purposeful creation of mental images. These findings are in line with studies on cognitive processes that have found that mental contrasting energizes goal pursuit and establishes strong associations between mental representations of the future and the obstacles, as well as between the mental representations of the obstacles and the means to overcome them (Kappes et al., 2012, 2013; Kappes and Oettingen, 2014).

This overlap between neurophysiological and cognitive approaches in research on intention formation corresponds with the overlap of these approaches observed in the intentional control of action by implementation intentions. Moreover, intention formation has been shown to impact the breadth of visual attention (Büttner et al., 2014). Using an eye-tracker, Büttner et al. (2014, Study 3) demonstrated that participants who had thought about how to act on a self-set goal (i.e., who were in an implemental mindset, which is active after an intention has been formed) subsequently focused on central objects in front of natural backgrounds (e.g., a train in front of mountains) more often than participants who had thought about whether they should commit themselves to a goal or not (i.e., who were in a deliberative mindset, which is active before an intention has been formed). Thus, the activation of cognitive procedures that deal with weighing the pros and cons of a potential goal was associated with a more balanced exploration of a visual scene, whereas the activation of cognitive procedures that deal with the translation of one’s intentions into action was associated with a visual exploration biased toward the central object.

### Conclusion

The present review examined the status quo of the physiological research on implementation intentions and highlighted potential directions for future research. Although the number of physiological studies on implementation intention effects is still small, these studies already provide new insights into the temporal distribution and spatial location of these effects. Moreover, the initial physiological findings are in line with behavioral research on cognitive processes and involving critical samples, as well as the assumption that implementation intentions indeed strategically automate the intentional control of action. These findings and recent advances in conceptualization, modelling, analysis, and physiological and cognitive methods are encouraging, and we hope to see more research linking behavioral, cognitive, and physiological perspectives that will further improve our understanding of how implementation intentions support action control.

## Conflict of Interest Statement

The authors declare that the research was conducted in the absence of any commercial or financial relationships that could be construed as a potential conflict of interest.
